# Beyond dormancy: organ-specific gene regulatory networks control winter development in peach buds

**DOI:** 10.1093/hr/uhaf310

**Published:** 2025-11-06

**Authors:** Justin Joseph, Giorgio Perrella, Riccardo Aiese Cigliano, Marco di Marsico, Monica Canton, Esther Carrera, Lucio Conti, Claudio Bonghi, Serena Varotto

**Affiliations:** Department of Agronomy, Food, Natural resources, Animals and Environment (DAFNAE) Agripolis, University of Padova, Viale dell’Università, 16, 35020 Legnaro (PsD), Italy; Department of Biosciences, University of Milan, Via Giovanni Celoria 26, 20133 Milan, Italy; Sequentia Biotech, Carrer Dr. Trueta 179, 3°5ª, 08005 Barcelona, Spain; Sequentia Biotech, Carrer Dr. Trueta 179, 3°5ª, 08005 Barcelona, Spain; Department of Agronomy, Food, Natural resources, Animals and Environment (DAFNAE) Agripolis, University of Padova, Viale dell’Università, 16, 35020 Legnaro (PsD), Italy; Instituto de Biologıa Molecular y Celular de Plantas (IBMCP), Consejo Superior de Investigaciones Cientıficas (CSIC), Universitat Politècnica de València, Valencia, Spain; Department of Biosciences, University of Milan, Via Giovanni Celoria 26, 20133 Milan, Italy; Department of Agronomy, Food, Natural resources, Animals and Environment (DAFNAE) Agripolis, University of Padova, Viale dell’Università, 16, 35020 Legnaro (PsD), Italy; Department of Agronomy, Food, Natural resources, Animals and Environment (DAFNAE) Agripolis, University of Padova, Viale dell’Università, 16, 35020 Legnaro (PsD), Italy

## Abstract

Bud dormancy in temperate perennials is often described as a stereotyped state of developmental repression triggered by environmental signals. Here, we investigate the development of vegetative buds in *Prunus persica* during the cold season to assess whether, like flower buds, they remain transcriptionally active. An integrated approach combining cytological analysis, hormone profiling, transcriptome sequencing, co-expression and gene regulatory network (GRN) inference, and *in vivo* interaction assays was used to compare bud types. Despite similar levels of abscisic acid and gibberellins during chilling accumulation, vegetative and flower buds displayed divergent transcriptional responses. Vegetative buds activated jasmonate- and photoperiod-responsive gene modules, while floral buds were marked by chilling-responsive modules regulated by SHORT VEGETATIVE PHASE 1 (SVP1). Bimolecular fluorescence complementation confirmed specific interactions between SVP1 and DORMANCY-ASSOCIATED MADS-box (DAM) proteins DAM3, DAM5, and DAM6. GRN analysis highlighted bud-specific combinations of DAM and SVP proteins, with DAM5 and DAM6 homodimers predominant in vegetative buds and DAM4 and SVP1/2 heterodimers dominant in flower buds. Our data revise the classical dormancy paradigm: flower and vegetative buds share hormonal trends yet deploy distinct MADS-box combinations to coordinate environment-dependent winter development. The organ-specific DAM/SVP circuitry uncovered here provides a new framework for mechanistic studies on cold mediated peach bud development.

## Introduction

Temperate deciduous fruit trees synchronize their developmental transitions with seasonal environmental changes, relying primarily on photoperiod (length of day, LOD) and temperature to regulate winter dormancy cycles and apical meristem activity [1]. As climate change alters seasonal temperature patterns, understanding the mechanisms underlying bud development has become central to predict phenological responses and ensuring productivity in fruit tree species [2, 3].

Dormancy, originally defined as the temporary suspension of visible meristematic growth [4], is classically categorized into three types: paradormancy, imposed by signals from other parts of the plant; endodormancy, controlled by bud internal factors; and ecodormancy, governed by unfavorable external conditions. This tripartite framework has been widely used to describe bud quiescence in woody perennials [5]. However, its direct application to fruit trees in the Rosaceae family remains problematic [6]. These species display considerable diversity in bud types—vegetative, floral, and mixed—each one with specific topological positions and developmental dynamics. As a result, the extent and timing of dormancy may vary significantly depending on the bud type, making a uniform classification inadequate [7, 8]. However, in Rosaceae species, the physiology of buds during the cold season remained anchored to the dormancy paradigm, although cytological and molecular evidence pointed out an active differentiation in apple and peach [9, 10].

In *Prunus persica*, flower and vegetative buds exhibit divergent behaviors [6]. Flower buds, which are lateral, continue to develop slowly through the winter, culminating in male and female gametophyte formation before bud break [11–13]. Vegetative buds—apical and responsible for shoot growth—are commonly assumed to remain dormant, but little is known about their development during the cold season [6]. Therefore, in peach, the coexistence of developing flower and quiescent vegetative buds under the same environmental conditions poses a fundamental question: do vegetative buds truly enter dormancy, or do they follow a different developmental program? Transcriptome analysis has been carried out only in flower buds in which MADS-box genes DORMANCY-ASSOCIATED MADS-BOX (DAM) and SHORT VEGETATIVE PHASE (SVP) play pivotal roles [12, 14, 15]. Both SVP and DAM genes are transcriptionally upregulated in the initial phase of chilling unit (CU) accumulation, and their expression level decreases toward the end of winter before flower bud break. Concomitantly to their upregulation DAM’s chromatin is enriched in trimethylation of lysine 4 in histone H3 (H3K4me3), a histone modification associated with active transcription [12, 14, 16]. Most functional studies to date have focused on vegetative buds in the poplar model system, where dormancy is marked by early meristem growth arrest and callose deposition, followed by cold-induced growth reactivation [17]. Whether the same regulatory logic applies to fruit trees—and whether DAM and SVP genes operate similarly in vegetative and flower contexts—remains poorly understood. Similarly, profiling of hormones combined with transcriptome approaches has been restricted to flower buds [18, 19]. To date, no comprehensive transcriptome or hormonal analysis of *Prunus persica* vegetative buds during the cold season has been reported.

In this study, we address this gap by investigating the development of vegetative buds in *Prunus persica* during autumn and winter. We combine cytological, hormonal, transcriptomic, and network-based analyses to explore whether vegetative buds, like their flower counterparts, remain active during chilling accumulation and how they integrate environmental signals to coordinate their development. We further examine the behavior of DAM and SVP transcription factors, assess their physical interactions through BiFC and protein docking, and reconstruct gene regulatory networks (GRNs) to define their bud-specific regulatory logic. By comparing flower and vegetative buds under identical environmental conditions, we aim to challenge the notion of dormancy as a stereotyped repressive state and provide new insights into organ-specific regulatory mechanisms in perennial species.

## Results

### Vegetative bud morphology

**Figure 1:**
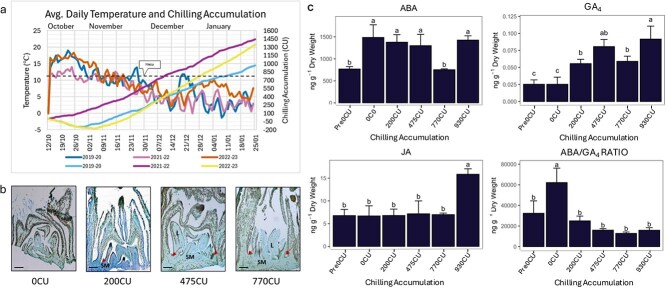
(a) Variation between weather patterns over the course of 3 years and the resulting variation in CU accumulation. The dashed line indicates the attainment of 770CU which denotes CU accumulation completion. (b) Longitudinal sections of vegetative buds stained with 0.1% Toluidine blue, showing the gradual increase in the meristematic region within the vegetative buds during the progression of CU accumulation. SM: Shoot meristem; L: leaflets. The arrows indicate the progressive addition of annular meristematic regions. Bars = 500 μm. (c) Variation of hormones with the accumulation of CU for ABA, GA_4_, JA and the ratio between ABA/GA_4_ in vegetative buds. Differences were considered significant at the *P* ≤ 0.05.

To visualize the morphological modifications occurring during the CU accumulation period (Fig. 1a) longitudinal sections of vegetative buds were observed under the light microscope at 0, 200, 475, and 770CU. Although organ-resolved estimates of CR are not available for ‘Fantasia’, vegetative and flower buds were sampled synchronously at equivalent CU levels according to the Utah model. This design has been adopted for the comparison between different bud types and is supported by the experimentally determined flower-bud CR of approximately 770 CU [20] and by reports of comparable CR ranges between flower and vegetative buds in other peach cultivars [21, 22]. Nevertheless, the lack of ‘Fantasia’-specific CR data for vegetative buds should be considered a limitation when interpreting organ-specific developmental dynamics. The vegetative buds underwent differentiation and elongation and showed an increase in the number of leaflets formed within the bud during the period of CU accumulation (Fig. 1b). The most significant differences can be observed after 475CU where there is an increase in the number of annular meristematic regions within the bud along with the elongation of leaflet. These observations indicate that vegetative buds are indeed active, and they continue to develop during winter. To facilitate interpretation, in presenting the results we consistently refer to chilling accumulation and cold development, without explicitly distinguishing endodormancy and ecodormancy stages.

### Quantification of hormones

Hormone quantification in vegetative buds was conducted for 6 timepoints. A one-way ANOVA test was carried out to identify the variation of hormonal content with respect to CU accumulation (Table 1) and the resulting trends are shown in Fig. 1c. Compared to hormone levels in flower buds [12], vegetative buds exhibited a comparable pattern in the accumulation of Abscisic acid (ABA) and active Gibberellin (GA_4_), as well as for the ABA/GA_4_ balance. However, the ABA and GA_4_ levels increased post CU accumulation. Jasmonic acid (JA) accumulation remained stable throughout most of the CU accumulation period but significantly increased after CU accumulation was completed.

**Table 1:** Flower and vegetative bud sampling calendar

**Timepoint**		**Length of day (hours)**	**Chilling accumulation (CU)**	**Sampling period**	**Date of sampling**
**Flower buds samplings**					
Pre0CU		12.85	0	B	8 September 2021
0CU		10.2	0	A	31 October 2019
200CU		9.42	200	A	18 November 2019
474CU		8.88	475	A	6 December 2019
770CU		8.74	770	A	27 December 2019
930CU		8.72	930	B	21 December 2021
**Vegetative buds samplings**					
Pre0CU		12.85	0	B	8 September 2021
0CU		10.2	0	A	31 October 2019
200CU		10.04	200	B	3 November 2021
475CU		8.88	475	A	6 December 2019
475CU		9.22	475	B	23 November 2021
770CU		8.74	770	A	27 December 2019
930CU		8.72	930	B	21 December 2021

The stable or transient accumulation of indol-3-acetic acid (IAA), GA₁, and cytokinins (CKs: dihydrozeatin, DHZ, isopentenyladenine, iP, trans-zeatine tZ) in vegetative buds during chilling accumulation suggests a minor regulatory role for these hormones (Fig. S1).

### Transcriptomes of the vegetative and flower buds during the period of CU accumulation

To study the transcriptome of vegetative buds and comparing it with the one from flower buds, high quality reads from RNA samples extracted at 0, 200, 475, and 770CU and two additional timepoints, one before and one after chilling accumulation for flower buds as well as vegetative buds, were mapped to the latest peach reference genome (*Prunus persica* v.2.5, NCBI). The previous reads for the flower buds from Canton *et al*. [12] were treated with the same preprocessing and mapping algorithm to maintain uniformity across the multiple datasets. The batch effect resulting from the combination of independent datasets was eliminated using the Arsynseq function from the NOISeq package (Fig. S2a, b, e, and  f). The resulting transcriptomes were evaluated by performing a principal component analysis (PCA) for the clustering of replicates from the respective timepoints. PCA indicated that the first two PCs explain most of the variance (79.1% for vegetative buds and 62.4% for flower buds), and samples from each time point are projected together (Fig. S2d and h). Filtering the DEGs using the Degust online platform for genes with FC >1.4 identified 8882 differentially expressed genes (DEGs) in vegetative buds and 8639 DEGs in flower buds throughout the different time points. 5913 DEGs were common in both transcriptomes (vegetative buds and flower buds), while 2969 and 2726 DEGs were unique in vegetative and flower buds, respectively (Fig. S3). The time-course analysis of CU accumulation (Fig. S3a) shows a gradual increase in the number of DEGs in both buds. In addition, the analysis revealed that the vegetative buds showed a higher upregulation than the flower buds, primarily during the initial period between pre-chilling and 0CU accumulation. Conversely, at 200CU and 475CU, both bud types do not show significant changes within the transcriptome. Subsequently, at 770CU a sudden increase of downregulated DEGs in vegetative buds is observed, whereas in flower buds there is a comparatively higher number of upregulated DEGs. At 930CU, there is a significant increase in the number of upregulated DEGs in flower buds, whereas the number of downregulated genes is almost identical between the two bud types. The Venn diagrams comparing the DEGs (Fig. S3b) from the two bud types throughout the entire time-course identify 710 DEGs that are differentially expressed in vegetative buds from beginning to end, whereas in flower buds there are 1041 DEGs.

**Figure 2:**
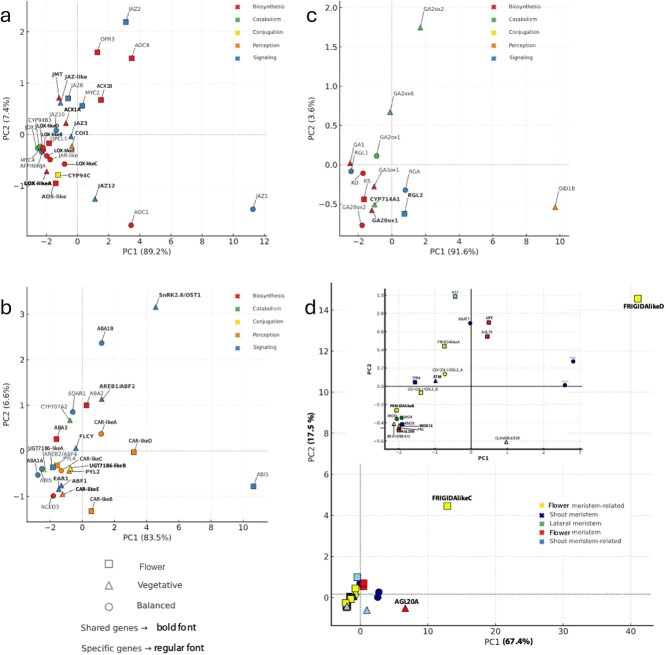
Principal component analysis (PCA) of gene expression patterns for ABA (a), GA (b), and JA (c) pathway genes, and meristem genes in flower and vegetative buds. Symbols indicate gene classification based on organ-biased expression: flower (open squares), vegetative (open triangles), and balanced (open circles). For Hormone-pathway genes are grouped according to functional category, as indicated by the accompanying legend in each panel. Categories include: biosynthesis, catabolism, conjugation, perception, and signaling. Meristem-related are grouped according to their functional class, as indicated by the legend in panel (d). Categories include flower-meristem related, shoot-meristem related, lateral meristem, flower-meristem identity, and shoot-meristem–related classes. Shared genes (expressed in both buds) are labeled in bold font, specific genes (organ-specific) in regular font. PCA was computed using shared genes, with specific genes subsequently projected into the same PCA space. Detailed procedures are described in Methods S3.

Considering that the hormone content between vegetative (Fig. 1c) and flower buds [12] remains very similar during the cold season, while their development diverges significantly, we performed a targeted PCA on differentially DEGs associated with ABA, GA, and JA pathways, as well as on genes involved in the specification and development of vegetative and flower meristems [23] (Table S2 and S3) (Fig. 2).

PCA of ABA-, GA-, and JA-related gene expression profiles revealed distinct spatial arrangements, although in all three cases, the first principal component (PC1) captured the major axis of organ-specific variation, as confirmed by its strong correlation with the differential expression coefficient condition (coef_condition) (Table S2). The second component (PC2) accounted for a smaller portion of variance and did not correlate with organ identity, but in some cases reflected functional or pathway-level diversity.

In the ABA PCA (Fig. 2a), PC1 explained 83.5% of the total variance and was strongly negatively correlated with coef_condition (*r* = −0.88), separating vegetative-biased genes (positive PC1 scores) from flower-biased ones (negative scores). Several transcriptional regulators involved in ABA signaling (*AREB1/ABF2* and *EAR1*) were enriched in vegetative buds, while *FLCY* and *PYL4* showed flowerl-biased profiles. A group of central ABA metabolism and signaling genes (*NCED3, PYL1, ABI5*) clustered near the origin, suggesting balanced expression between organs. Vegetative-specific genes projected more diffusely along PC1, indicating transcriptional heterogeneity within vegetative tissues. PC2 (6.6% variance) did not associate with expression bias or timepoint and appeared to reflect minor variation among shared genes. No marked outlier was observed in this dataset.

In the GA PCA (Fig. 2b), PC1 accounted for 91.6% of variance and showed a strong positive correlation with coef_condition (*r* = +0.92), inverting the directionality observed for ABA and JA. Genes involved in GA biosynthesis (*GA20ox2, GA1,* and *GA3ox*) and catabolism (*GA2ox2, GA2ox6*, and *CYP714A1*) were mapped to the vegetative side, while RGL2, a DELLA protein, displayed a flower signature. The GA receptor-encoding gene *GID1B* occupied an extreme positive position on PC1, suggesting distinct vegetative-specific regulation or feedback sensitivity. PC2 (3.6% variance) did not reflect organ bias or expression strength.

In the JA PCA (Fig. 2c), PC1 explained 89.2% of variance and was strongly negatively correlated with coef_condition (*r* = −0.83), separating vegetative-biased (*LOX-likeA, JMT,* and *ACX1A*) from flower-biased (*ACX1B,* and *AOS-like*) genes. PC2 (7.4% variance) captured pathway-internal diversity, separating signaling components (e.g., JAZ genes) from biosynthetic ones, but without clear association to time or organ. *JAZ1* emerged as a strong outlier in the PCA space. Although statistically classified as ‘balanced’ due to high within-group variance and a non-significant expression differential, it showed consistently higher expression in flower buds. Among all JA-related genes, it was also one of the most highly expressed overall. Its extreme position along PC1 reflects this combination of high expression and organ-skewed pattern and supports its known function as a core jasmonate repressor potentially involved in modulating flower bud development.

**Figure 3:**
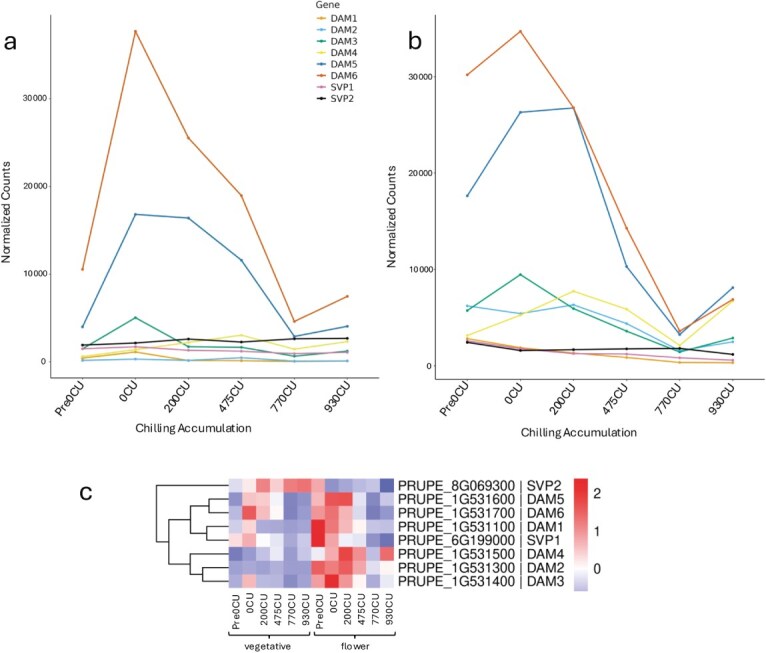
Temporal expression profiles of *DAM* and *SVP* genes during chilling accumulation. Temporal expression profiles (normalized counts) of DORMANCY-ASSOCIATED MADS-box (*DAM1–DAM6*) and SHORT VEGETATIVE PHASE-like (*SVP1, SVP2*) genes in vegetative (a) and flower (b) buds across six chilling timepoints (from 0 to 930CU); (c) Heatmap of Z-score-transformed expression values of DAM and SVP genes in both bud types.

In the PCA of meristem-related genes (Fig. 2d and Table S2), a clear separation emerged between central conserved meristem regulators and peripheral genes associated with bud-specific identity. Core shoot meristem genes, including *WOX* and *KNAT*, clustered centrally, reflecting their stable expression across bud types. In contrast, genes marking vegetative or flower fate were distributed along divergent axes, consistent with their functional specialization. Among flower meristem identity regulators, LFY, AGL20B, and AGL79 formed a coherent cluster in flower-biased positions, indicating early activation of reproductive programs. Conversely, meristematic-specific markers such as STM and WOX3 were positioned along the vegetative axis, supporting their role in maintaining vegetative identity during chilling.

The FRIGIDA-like gene family members also displayed divergent patterns: *FRIGIDA-like B, C*, and *D* were exclusively expressed in flower buds, yet only *FRIGIDA-likeD* showed a strong flower association in the PCA, with clear separation along PC2. *FRIGIDA-likeA*, while expressed in both bud types, was enriched in flower buds, suggesting a broader regulatory function with flower bias.

Interestingly, genes canonically associated with flowering induction, such as *AGL20A* and the *CO/COL1/COL2-A* displayed expression patterns biased toward vegetative buds or balanced across bud types, respectively. This observation suggests that these regulators, while linked to flower transition in annual models, may also participate in modulating photoperiodic or hormonal responsiveness in vegetative buds of perennials, potentially maintaining developmental plasticity under chilling conditions.

### DAM and SVP gene dynamics in flower and vegetative buds

Examining the expression profiles of the *DAM* genes in our flower and vegetative bud dataset revealed a decreasing general trend during CU accumulation in both bud types (Fig. 3a and b), with *DAM5* and *6* remaining highly expressed over CU accumulation. In vegetative bud, the other DAMs are expressed at a low level and steady state, while in flower bud *DAM2*, *3* and *4* showed a higher expression during the early phase of CU accumulation (Fig. 3c). Notably, *DAM4, 5*, and *6* exhibited a reactivation of transcription accumulation at the late stage of 930CU.

In our previous studies we identified an SVP-like gene transcript (*Ppe*6G199000) belonging to the SVP1 clade and expressed in flower buds before CU accumulation and downregulated during winter [12]. A novel SVP gene transcript was identified in the current vegetative bud dataset (*Ppe*8G069300) that belongs to the SVP3 clade [24]. The expression level of the two SVP-like genes is much higher in vegetative buds, and their expression trends are also divergent (Fig. 3a–c). While the previously identified *PpeSVP1* gene undergoes a rapid decline in expression levels with the progress of CU accumulation in flower buds [12], in the vegetative buds *PpeSVP1* maintains a steadier expression level. *PpeSVP2* is highly expressed in flower buds in prechilling and then its level slowly decreases. In vegetative buds the expression of this gene follows an opposite trend with the expression upregulated over CU accumulation (Fig. 3a). These results indicate that except for *DAM5* and *DAM6*, DAMs and SVPs genes are differentially regulated during winter development in vegetative and flower buds.

### Weighted gene co-expression analysis of DEGs

**Figure 4:**
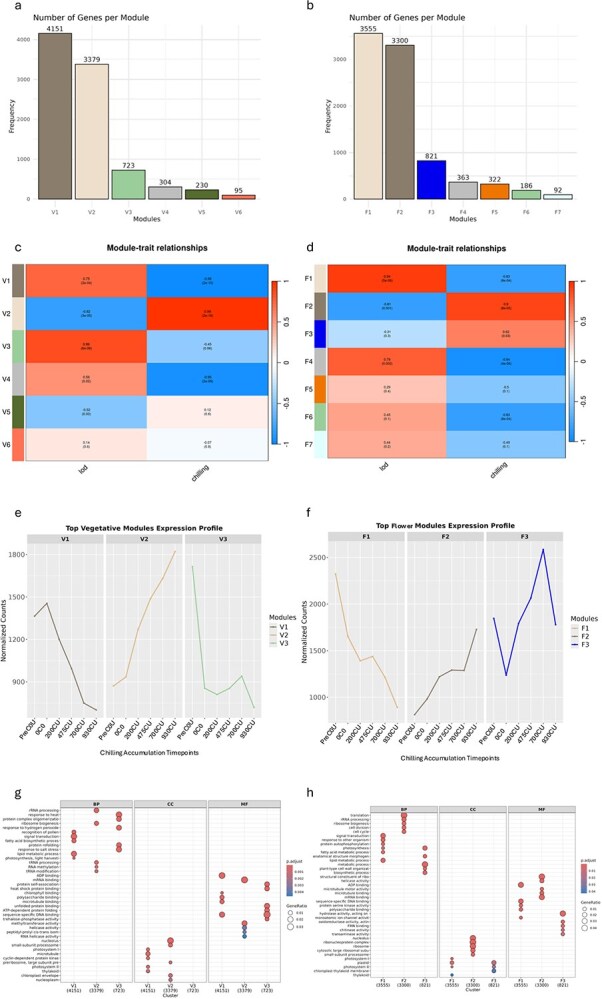
WGCNA in vegetative and flower buds. Panels a, b: the distribution of DEGs in the different correlation modules (V, vegetative buds; F; Flower buds); c, d: Module-environmental cues (Length Of Day, LOD; CU accumulation; CHILLING). e, f: Expression pattern of the co-expressed genes in selected modules. Panels g, h: GO enrichment of the genes in the selected modules.

The WGCNA package was used to conduct a gene co-expression analysis and clustering of the identified DEGs. DEGs with a fold change of 1.5 and FDR value lower than 0.01 were selected as the input for the network analysis. The network analysis was used to correlate the DEGs against two environmental cues having opposite trends during winter: CU accumulation (CHILLING) increased (Fig. 1) while the length of day (LOD) decreased (Fig. S4). Both vegetative and flower buds produced 6 and 7 modules (MEs; Fig. 4a and b and Tables S3 and S4).

**Figure 5:**
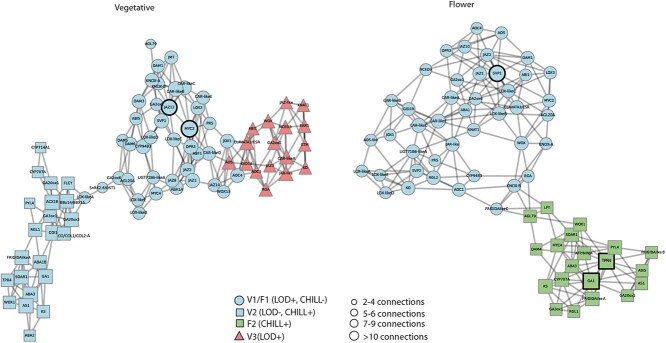
Co-expression networks of hormone- and meristem-related genes in vegetative (V) and flower (F) buds. Weighted gene co-expression networks were constructed using genes from WGCNA modules significantly associated with environmental cues during the winter period. The left panel represents the network for vegetative buds, including modules V1, V2, and V3), while the right panel shows the floral bud network, comprising modules F1 and F2. Each node corresponds to a gene, and edges represent strong pairwise correlations (Pearson’s *r* ≥ 0.75). Only the top five most correlated genes were retained for each node to improve network readability. Node shape identifies the module to which each gene belongs as indicated in the legend (circles, squares, and triangles). Gene modules differ in their regulatory association with environmental factors: modules V1 and F1 are positively correlated with photoperiod (LOD) and negatively with chilling (mixed regulation); module V2 is negatively correlated with LOD and positively with chilling (also mixed); module F2 is regulated by chilling only (positive correlation); and module V3 is associated exclusively with LOD (positive correlation). Node size is proportional to the number of edges (2–4, 5–6, 7–9, or ≥10), indicating the degree of co-expression; nodes with a bold outline represent genes with ten or more connections.

Correlation analysis between MEs and transcriptome profile (Fig. 4c and e) revealed significant correlations in vegetative buds for three modules (V1, V2, and V3) with LOD and 2 modules (V1 and V2) with CHILLING. Specifically, the expression gene in V1 was positively correlated with LOD (*r* = 0.75, *P* = 3e^−04^) and negatively with CHILLING (*r* = −0.96, *P* = 2e^−10^), conversely, V2 showed an opposite correlation to the environmental cues (*r* = −0.82, *P* = 3e^−05^; *r* = 0.99, *P* = 2e^−16^ for LOD and CHILLING, respectively). For V3, a positive correlation (*r* = 0.86, *P* = 6e^−06^) was found only for LOD, suggesting a distinct regulatory identity. For flower buds (Fig. 5D and F), the analysis highlighted that a ME (F1) and two MEs (F1 and F2) were significantly correlated to LOD and CHILLING, respectively **(**Fig. 4d). F1 was positively correlated with LOD (*r* = 0.94, *P* = 5e^−06^) and negatively with CHILLING (*r* = −0.83, *P* = 8e^−04^), while F2 was positively correlated with CHILLING (*r* = −0.9, *P* = 8e^−05^). Interestingly, considering the number of genes in each significant ME in the two bud types, the environmental cues differently impacted gene expression (e.g., LOD in vegetative buds and CHILLING in flower buds). In flower buds, more than 3000 genes (F2) are regulated only by CHILLING. Although the F3 module contained 821 DEGs, no significant correlation with environmental cues was observed (Fig. 4d). GO functional analysis (Fig. 5g and h) highlighted active transcriptional programs in both vegetative and flower buds, with common enrichment in categories such as photosynthesis, signal transduction, and stress response. In flower buds, the enrichment in defense-related processes and ribosomal structural components suggests a high biosynthetic activity consistent with flower differentiation. ADP binding and chlorophyll binding were shared between both bud types, possibly reflecting metabolic continuity or plastid-mediated signaling. Vegetative buds showed specific enrichment in helicase activity, indicative of ongoing chromatin or RNA remodeling, while flower buds were enriched in DNA-binding and kinase activity, in line with transcriptional reprogramming and organ identity regulation.

To investigate the transcriptional coordination of hormone- and meristem-related genes in response to environmental cues, we reconstructed co-expression networks from selected WGCNA modules significantly correlated with CHILLING and LOD in flower and vegetative buds (Fig. 5). The networks were restricted to DEGs functionally annotated as hormone-related (ABA, GA, JA) or involved in meristem identity and maintenance (see Tables S2 and S3), excluding hubs identified purely through topological ranking. This targeted strategy avoids centrality-driven bias and provides a biologically informed map of regulatory integration across bud types.

In vegetative buds, the network revealed three discrete clusters corresponding to modules V1, V2, and V3. The V1 module formed a most connected core including key meristem regulators (WOX13 and KNOX), GA catabolism (GA2ox and GA2ox6) and their negative regulators (RGL2), and key JA signaling components (MYC2 and JAZs), suggesting that this module may act as a day length-responsive network that buffers vegetative identity and restrains premature growth during winter. Notably, DAM1 was embedded within this cluster but did not emerge as a central hub (Table S5). The V2 cluster, under opposing environmental regulation, showed lower overall connectivity, with ABA signaling repression (FLCY), JA (ACX1B and COI1), and GA (GA20ox and KS) biosynthesis and signaling. V3, regulated exclusively by LOD, contained meristem genes such as *STM, KNAT1,* and *CLAVATA3/ESR*, highlighting a coherent transcriptional program distinct from the mixed-regulation modules.

The flower bud network included modules F1 and F2. F1 reproduced the vegetative core with high modularity and integration of hormonal signaling pathways. A key feature of this module was the emergence of SVP1 as a prominent connected hub (connected degree ≥10, Table S6), reinforcing its role as a central integrator in flower bud development. SVP2 was present but less connected. The F2 cluster, exclusively responsive to CHILLING, included most flower meristem genes (*FRIGIDA-like, LFY, AGL79*) and displayed low overall connectivity, suggesting a specialized yet decentralized module. *GA1* and *TPR4*, encoding an ent-copalyl diphosphate synthase involved in GA biosynthesis [25] and a member of the TOPLESS family of corepressors [26], respectively, showed the highest connectivity, indicating their potential role in modulating cold-induced flower differentiation. Overall, these data suggest that jasmonate signaling is prominently embedded in LOD-responsive vegetative modules, while ABA and GA pathways contribute to both shared and organ-specific programs. DAM genes, although environmentally responsive, do not play central roles in co-expression networks.

### BiFC assay identifies interactions among SVP and DAM genes

Although SVP and DAM genes did not emerge as central regulators within the co-expression networks—particularly in flower buds—their roles in dormancy and developmental transitions prompted us to investigate their potential interactions with other developmental regulators in vegetative buds. We performed a BiFC assay on *N. benthamiana* leaves to test for physical interactions between SVP1 and a set of 11 candidate target genes, previously identified in Arabidopsis SVP interactomes and found among the DEGs in vegetative buds (Table S7).

Of the 11 targets SVP1 was found to interact with 3 which were all DAM namely DAMs 3, 5, and 6. The positive and negative controls were chosen based on the literature on related species. The DAM5-DAM6 pair was confirmed as positive interaction, while DAM6-DAM3 did not seem to interact. The SVP1 protein was found to interact with all the three DAM (3, 5, 6) proteins. To further validate and document the interaction pattern, we expanded the assay to include the full set of candidate gene pairs. As shown in Fig. S5, nuclear YFP fluorescence (indicative of a positive interaction) was observed only for SVP1 interactions with DAM3, DAM5, and DAM6. No fluorescence signal was detected for SVP co-expressed with the other candidate regulators (PILS7, CIB8, PIF8, AP2-2, ERF1, ERF2, and AUX/IAA rep), confirming the specificity of SVP1–DAM interactions in this context. RFP signal is used as positive control of *N. benthamiana* infiltration.

**Figure 6:**
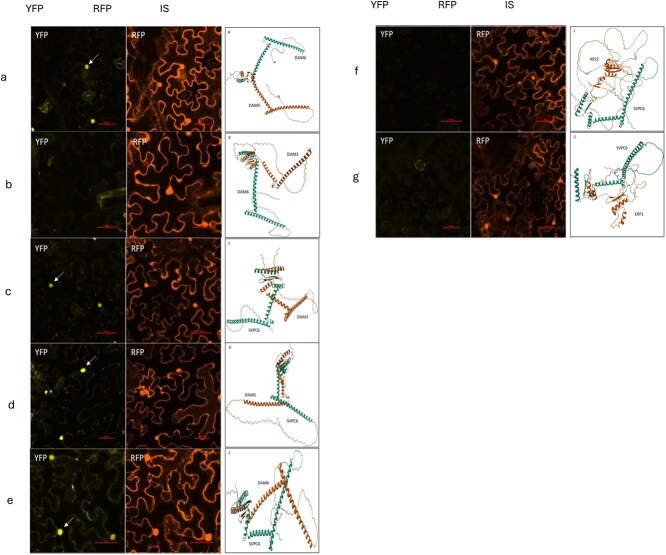
Each panel displays the YFP and RFP channels from the confocal microscope as well as the in-silico modeling of the protein-pair interaction (IS). The RFP signal represents the internal control for transformation. Nuclear fluorescence highlighted with an arrow in the YFP channel denotes a positive interaction. a and b represent the positive (DAM5-DAM6) and negative (DAM6-DAM3) controls. Panels c, d, and e represent the positive interactions between SVP-DAM3, SVP-DAM5, and SVP-DAM6, respectively, while panels f—AP22 and g—ERF1 did not show interactions with SVP1.

To better understand the selective interaction between DAMs and SVP1, docking studies were conducted using the Z-DOCK online platform. Individual protein structures predicted using the Alphafold2 software with high confidence scores were selected and used to conduct pairwise and multimeric docking studies. While there was the presence of some false positives, most of the results coincided with the findings from the BiFC assay (Fig. 6a).

Together, the BiFC assay and protein–protein docking analysis confirmed a selective physical interaction between SVP and members of the DAM subfamily, with no detectable interactions involving other candidate regulators tested.

### DAM/SVP gene regulatory networks in flower and vegetative buds

Three distinct gene regulatory networks (GRNs) were reconstructed to identify DAM/SVP transcriptional targets in peach buds: one specific to flower buds, one to vegetative buds, and one comprising shared targets. Networks were inferred using the GENIE3 algorithm and integrated with WGCNA module assignments and BiFC-based interaction validation. To define high-confidence regulatory targets, we applied a filtering step based on the distribution of GENIE3 importance scores. Empirical thresholds were set at 0.30 for flower buds, 0.36 for vegetative buds, and 0.27 for shared targets, in agreement with dataset-specific distributions and literature-supported cutoffs for biologically meaningful interactions [27]. All selected targets, along with their regulatory scores, are listed in Tables S8–S10, and the distribution of importance values is shown in Fig. S6. Because targeted analyses (Figs 2 and 5) were restricted to hormone- and meristem-related gene subsets, a limited overlap with GRN-derived targets was expected, allowing the identification of additional regulatory components beyond the original selection scope.

**Figure 7:**
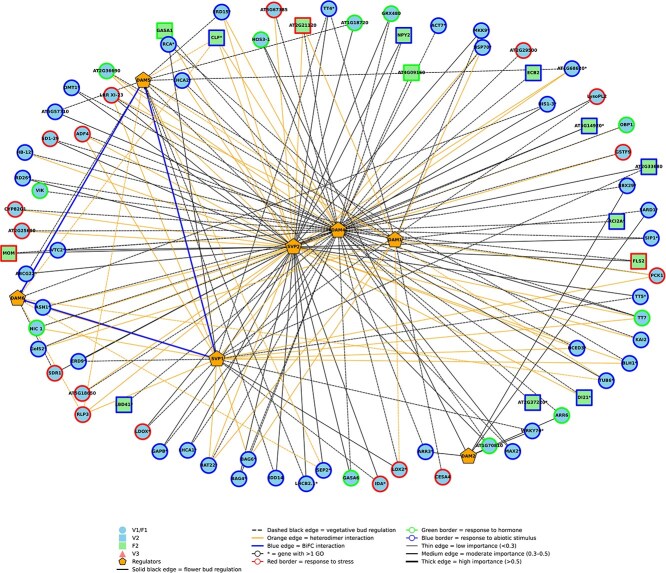
Shared Gene regulatory network (GRN) comprising predicted targets for DAM and SVP-like transcription factors in peach buds. Nodes represent target genes (circles, squares, triangles) or transcriptional regulators (pentagons). Node shapes indicates WGCNA module membership: circles (F1 and V1), squares (V2), squares (F2), and triangles (V3). Node borders denote Gene Ontology (GO) categories, as labeled in the legend:response to abiotic stimulus, response to stress, and response to hormone. Asterisks (*) indicate genes annotated in multiple GO categories. Only targets with significant GO annotation are shown.. Edges represent regulatory interactions inferred by GENIE3: solid edges flower-specific regulation, dashed edges indicate vegetative-specific regulation. Additional edges types represent distinct interaction modes defined in the legend; including heterodimer-based regulation, and BiFC-validated physical interactions. Edge thickness reflects GENIE3 importance scores: thin (<0.3, weak), medium (0.3–0.5, moderate), thick (>0.5, strong). Full interaction data and GO assignments are provided in Table S8.

The shared GRN (Fig. 7) comprises 309 target genes regulated by DAM and SVP-like transcription factors in both flower and vegetative buds. Although these regulators are expressed in both bud types, more than 85% of the shared targets (265 out of 309) are regulated by different combinations of transcription factors, indicating that transcriptional control is largely bud-specific despite overlapping target identity. In vegetative buds, regulation predominantly involves DAM5 and DAM6 homodimers, as supported by BiFC assays (Fig. 6), whereas in flower buds, DAM–SVP heterodimers are more frequently predicted. Among these targets, 93 genes are annotated with GO terms related to environmental response—namely response to hormone, response to stress, and response to abiotic stimulus—and are visualized in the network. Several ABA-related genes within this subset—such as *NCED3* (9-cis-epoxycarotenoid dioxygenase) and *PYL4*—were previously noted for their differential expression (Fig. 2a) and are now identified as predicted DAM/SVP targets. Although these genes are included in the WGCNA co-expression modules (Fig. 5), they are not connected to DAM/SVP regulators within the co-expression network, suggesting a regulatory relationship not mediated by co-expression. In contrast, other targets were identified exclusively through GRN inference, such as *GASA1* (*PRUPE_1G341800*), *GASA6* (*PRUPE_1G341900*), and *CLF* (PRUPE_7G130900). *GASA1* and *GASA6* belong to a family of gibberellin- and ABA-responsive genes previously shown to be expressed in dormant buds of pear (*Pyrus pyrifolia*) and implicated in the hormonal regulation of mixed bud dormancy release [28]. *PRUPE_7G130900* encodes a Polycomb-group gene homologous to Arabidopsis CLF, a key epigenetic regulator of flowering [29]. GRN analysis revealed that these genes are subject to distinct regulatory inputs in flower and vegetative buds: GASA1 and GASA6 are predominantly targeted by DAM5 and DAM6 homodimers in vegetative buds, whereas in flower buds they are regulated by DAM–SVP heterodimers. Similarly, *PRUPE_7G130900* is targeted by different DAM/SVP combinations depending on the bud type. This bud-specific regulatory pattern supports the hypothesis that these genes contribute to divergent developmental trajectories in flower and vegetative meristems.

A distinct regulatory architecture was predicted for flower buds (Fig. S7), comprising 618 candidate targets, 103 of which are annotated with GO terms. Among the 103 visualized targets, many were annotated with multiple categories. The most frequent annotations corresponded to developmental process (96 genes) and multicellular organism development (87 genes), indicating that the flower GRN captures a regulatory framework linked to reproductive fate and meristem identity. Notably, 97 of these targets were identified exclusively through the GRN approach, highlighting its added value in uncovering transcriptional components potentially missed by expression- or pathway-driven filters.

A closer inspection revealed that flower-specific targets are primarily regulated by DAM4 homodimers and SVP1/2, often in combination. *SEP1* and *SPL9* (*PRUPE_1G290500*, *PRUPE_6G256300*), both assigned to module F2 (chilling-responsive), were predicted to be regulated by DAM4 homodimers. *SEP1* encodes a class E MADS-box factor, while SPL9 mediates the flower transition [30]. The nucleolar gene *YAO* (*PRUPE_4G089600*), assigned to the category multicellular organism development, was also identified as a target of SVP-like homodimers consistent with its role in early microgametogenesis, as previously demonstrated in Arabidopsis [31].

The GRN specific to vegetative buds (Fig. S8) revealed 838 candidate targets, 142 of which are annotated with GO categories. As many targets are associated with more than one GO term, we focused on the most frequent functional classes, which included developmental process (142 genes), plant organ development, and post-embryonic development. Together, these categories reflect regulatory activity linked to meristem identity, organ patterning, and dormancy transitions. As observed for flower buds, most vegetative-specific targets (134 out of 142) were identified through GRN analysis. Importantly, vegetative-specific targets are predominantly regulated by DAM5 and DAM6 homodimers. We focused on representative genes involved in vegetative identity and polarity control. *KANADI1* (*PRUPE_5G223700*), assigned to module V3, was identified as a putative target of DAM6 homodimers. In Arabidopsis, KAN1 contributes to abaxial identity and meristem boundary maintenance [32]. Interestingly, the vegetative GRN includes an epigenetic regulator, EMF1, a Polycomb-associated protein known to repress the flower program [33], as a candidate target within this module.

## Discussion

Our findings indicate that peach vegetative buds, similarly to flower buds, slowly continue their development during winter. Histological observations revealed a gradual increase in the meristematic region within these buds, supporting the idea of vegetative buds as condensed, telescopic forms of branched structures and that their winter progression involves active morphogenesis rather than passive tissue swelling or metabolic priming. In Rosaceae, similar cytological observations on bud development were recently reported on apple terminal buds exposed to forcing conditions, from pre-chilling to before bud break [9].

### Hormonal integration: Interpreting ABA/GA balance in divergent contexts

To our knowledge, this is the first report about vegetative hormone content and transcriptome characterization in *Prunus persica* vegetative buds. Although ABA and GA₄ levels remain similar in both flower and vegetative buds during CU accumulation (Fig. 1c; [12]), their transcriptional outputs diverge substantially, indicating that bud fate is shaped not by hormone content alone, but by how hormonal signals are interpreted in different developmental contexts. In vegetative buds, ABA signaling appears transcriptionally active and stable. Genes involved in ABA perception and signaling—such as *PYL2*, *AREB1/ABF2*, and a CAR-like receptor (*PRUPE_3G162700*)—show consistent vegetative-biased expression (Fig. 2a), in line with ABA’s well-established role in restricting premature outgrowth [34]. Notably, the expression of *FLCY*, a farnesylcysteine lyase, is also upregulated in vegetative buds. This enzyme degrades farnesylcysteine, a known inhibitor of protein prenylation involved in ABA signaling [35]. In *Arabidopsis*, overexpression of *FLCY* confers ABA hypersensitivity, suggesting a buffering mechanism that modulates ABA responsiveness. *FLCY* is co-expressed with meristem regulators such as WOX13 STM genes in *Prunus* [36]. GA-related genes displayed a balanced expression profile: the biosynthetic gene *GA20ox1*, the catabolic gene *GA2ox6*, and the DELLA repressor *RGA* were all moderately and stably expressed (Fig. 2b). Rather than indicating shutdown of the GA pathway, this suggests a finely tuned equilibrium between biosynthesis, deactivation, and signal repression—maintaining the meristem in a growth-competent yet restrained state.

In flower buds, ABA-related genes such as *PYL4* and *ABI5* were co-expressed with flower meristem identity regulators including *LFY*, *AGL20B*, and *AGL79* (Fig. 3, [37]), suggesting a role for ABA in fine-tuning the timing of flower differentiation rather than repressing it. Although in Arabidopsis *ABI5* is classically associated with flowering inhibition, its activity may be modulated by repressors such as *ABI2*, facilitating flower transition through hormonal feedback loops [38].

Consistent with this interpretation, ABA and GA pathway genes in vegetative buds displayed remarkably stable expression between 200 and 475CU. Genes such as *PYL2*, *AREB1/ABF2*, *GA2ox6*, *GA20ox1*, and *RGA* maintained consistent levels across this phase (Table S1). This transcriptional plateau parallels the global expression pattern (Fig. S3A), suggesting a buffered regulatory state: while not inactive, the buds are maintained in a poised configuration where growth programs are preserved but not yet reactivated. A similar stability in ABA biosynthetic (*NCED*) and catabolic (*CYP707A*) gene expression was reported in peach by Wang *et al*. [39]**,** who interpreted it as part of an ABA-dependent mechanism maintaining dormancy. However, our data point to a more flexible scenario in which ABA signaling constrains visible growth but coexists with meristematic activity—reflecting a modulatory, rather than strictly repressive, role in both bud types.

### Jasmonate signaling and environmental integration via WGCNA modules

Photoperiod reduction and temperature decline have long been recognized as key environmental conditions promoting the onset of bud dormancy in perennial species [1].

In vegetative buds, the co-expression network reveals a robust integration of JA-related genes within the LOD-responsive module V1. Key components such as *MYC2* and several *JAZ* repressors are tightly co-expressed with meristem regulators including *WOX13* and *KNOX-like* genes, forming a coherent module likely involved in buffering vegetative identity during winter. This configuration aligns with the known role of MYC2 in restricting meristem activity and enhancing stress resilience under cold and photoperiodic cues [40, 41].

However, this apparent repression does not correspond to meristem arrest. The enrichment of *GA20ox* and *COI1* in V2 suggests that buds retain the ability to perceive and modulate GA and JA signaling inputs, potentially enabling a progressive reactivation phase. Meanwhile, the presence of *STM, KNAT1, and CLAVATA3/ESR* in V3 underscores the persistence of a core meristematic framework, even in the absence of visible growth.

In flower buds, the F1 module mirrors the vegetative core but with *SVP1* assuming a highly connected hub role, as previously reported in *Prunus mume* [42] and *Malus domestica* [43]. Within module F2, *TPR4* emerges as a key node alongside *GA1*, whereas in vegetative buds it is only marginally connected. Nonetheless, its interactions with *GA1*, S*OAR1*, and *ABA3* are conserved across both networks, suggesting a shared role in modulating hormone-related transcriptional responses. In flower buds, however, *TPR4* [26] expands its connectivity to include JA-related repressors such as *NINJA* and *MYC4* indicating an organ-specific recruitment into the JA signaling module [44]. This configuration reflects a poised transcriptional state—active but restrained—consistent with a dynamic balance between inhibitory and permissive signals, integrating hormonal cues such as ABA, GA, and JA in a stage-specific manner [45].

Three *FRIGIDA-like* genes [46] are present in WGCNA networks (Fig. 5) and although all show floral-specific expression patterns (Fig. 2d), one of them belongs to the V2 module. These results point towards a possible diversification of FRIGIDA-like role during bud growth, and it would be interesting to determine whether and how they are involved in regulating chromatin dynamics at specific target loci during winter development in vegetative and flower buds.

Across both networks, DAM genes remain topologically marginal, reinforcing the notion that their regulatory activity is not mediated through direct co-expression with hormone- and meristem-related targets. Thus, while *DAM5* and *DAM6* are responsive to chilling and included within relevant modules (Tables S3 and S4), their peripheral position within the co-expression graphs suggests that WGCNA cannot capture their role in winter development.

### GRNs differentiate regulatory logic: DAM/SVP complexes orchestrate divergent outcomes

Quesada-Traver *et al*. [47], propose that DAM activity adjusts developmental progression in a tissue-specific manner, modulating meristem behavior in response to cold rather than enforcing a uniform dormant state. Our GRN analysis reinforces this view by showing that DAM transcription factors engage distinct sets of targets in flower and vegetative buds, and that even shared targets are regulated via different DAM combinations depending on organ type (Fig. 7, Figs S7 and S8). For example, *GASA1* and *GASA6* are targeted by DAM5/6 homodimers in vegetative buds but by DAM–SVP heterodimers in flower buds. This illustrates how regulatory context—rather than target identity alone—shapes functional outcomes. The plasticity of these interactions aligns with the known roles of GASA proteins as context-dependent effectors of meristem activity, redox homeostasis, flower transition, and hormone crosstalk [48]. Another interesting example of differentiated regulatory activity concerns PRUPE_7G130900, the putative orthologue of PRC2 catalytic enzyme CURLY LEAF (CLF) responsible for spreading of the repressive mark H3K27me3 in plant chromatin [49], which is targeted by different DAM/SVP combinations in flower and vegetative buds. This observation indicates that in flower and vegetative buds, different pathways might be involved in establishing and propagating repressive chromatin domains in response to endogenous and environmental cues.

In flower buds, DAM4 appears to act in combination with SVP proteins to regulate *SEP1, SPL9,* and *YAO*, all known to function in flower commitment and gametogenesis, respectively [30, 31]. The sustained regulation of these targets after flower meristem specification suggests that DAM/SVP complexes may buffer gene expression, synchronizing developmental progression with chilling accumulation. The presence of SVP2-specific targets such as *YAO* further supports the idea that SVP2, within flower buds, contributes to regulatory refinement downstream of tissue identity. Amongst predicted DAM4-specific targets there is the callose synthase (CSLC5, Fig. S7). Our previous investigations on peach flower buds indicated the upregulation of genes for callose synthesis and increase in callose deposition in flower buds at the beginning of CU accumulation [12]. Callose synthesis and deposition for closing plasmodesmata and isolating the bud from the plants is one of the first responses of hybrid poplar vegetative buds to the signaling cascade that promotes growth arrest at the beginning of dormancy [17]. However, in peach vegetative buds, we did not observe any upregulation of callose synthesis genes, suggesting that callose deposition either occurs before the initial timepoints of our investigation or does not occur. Based on all these results, it can be speculated that callose synthesis might be more related to the differentiation of reproductive tissue than cellular communication. This interpretation is supported by recent spatial and single-cell transcriptomic data in *Prunus mume* [13], which show that callose synthase genes (CALS1, CALS7) are weakly expressed in the central floral domain suggesting active plasmodesmata and unrestricted cell-to-cell communication within floral tissues.

Vegetative buds (Fig. S8) exhibit a broader and less centralized regulatory structure, with DAM5 and DAM6 homodimers as dominant regulators. This view complements previous work showing organ- and tissue-specific roles of DAM6. Zhao *et al*. [15] demonstrated in floral buds that PpDAM6 regulates CR through ABA biosynthesis (PpNCED1) and callose deposition (PpCALS1/2), validated by functional assays. More recently, Zhao *et al*. [13] report that DAM6 expression is spatially restricted to outer tissues of flower buds with putative vegetative-like identity and excluded from the central floral domain. Together with our GRN analysis, these findings highlight DAM6 as a modular and pleiotropic regulator that assembles into distinct complexes depending on bud type and tissue context (e.g., DAM6–SVP or DAM6–NCED1/CALS in floral buds versus DAM6 homodimers in vegetative buds), thereby conferring organ-specific developmental outputs. Notably, only the vegetative GRN includes EMF1, a Polycomb-associated epigenetic repressor of flower programs [33]. This exclusivity suggests that vegetative buds utilize both transcriptional and epigenetic mechanisms to maintain developmental flexibility and prevent premature commitment.

Our findings support a revised model of dormancy in which DAM and SVP proteins act not as rigid repressors but as flexible transcriptional modulators operating within bud-specific regulatory landscapes. To be confirmed and to identify other molecular master regulators involved in cold-induced bud development this model needs further investigations. Although biotechnological functional validation approaches in peach remain technically immature—a robust and reproducible genetic transformation system in this species is still lacking [50] and ChIP experiments for target prediction requires antibodies, unavailable so far, with very high specificity and affinity for the native proteins—our integrative strategy offers the most comprehensive framework currently available.

## Materials and methods

### Weather records

Daily temperature readings were recorded from the Agenzia Regionale per la Prevenzione e Protezione Ambientale del Veneto (ARPAV) online portal (https://www.arpa.veneto.it/dati-ambientali/dati-storici) and maintained for the duration of October 2019 to January 2020 [12] and October 2021 to January 2022. For the same periods, to maintain a record of the length of the day, the daily sunrise and sunset times were obtained from online databases for the Region of Veneto (Fig. S4).

### Sampling of vegetative and flower buds

Vegetative and flower axillary buds from 12-year-old peach trees of the Fantasia (FAN) genotype, grown on the ‘Lucio Toniolo’ experimental farm at the University of Padova were collected in 2019–20 (Year A) and 2021–22 (Year B). To avoid positional variance, only axillary bud triplets were sampled, consisting of a central vegetative bud flanked by two lateral flower buds. Terminal buds were excluded, as they are exclusively vegetative and display distinct chilling and heat requirements compared to axillary buds [21]. Timepoints of collection were determined according to the UTAH model of accumulation of CU, calculated as described by Richardson *et al*. [51]. In both years, at 0, 200, 475, 770CU vegetative and flower buds were collected from three or four tree to form biological replicates. In Year B sampling was extended to include an early stage prior to chill accumulation (pre0CU), defined as the last collection date before the first effective CU were accumulated, plus an additional timepoint (930CU) after the fulfillment of Fantasia CR [52].

Specific calendar dates for each collection are provided in Table 1. The buds were immediately cleaned to remove the external scales, frozen in liquid nitrogen, and stored at −80°C.

### Vegetative bud fixation for cytological observation

Vegetative buds collected during the two sampling periods were fixed in 0.1 M phosphate buffer solution with 4% paraformaldehyde and embedded in paraffin (Paraplast Plus, Sigma-Aldrich) and sectioned at 10 to 12 μm using a microtome (RM2135; Leica, Germany). Sections were mounted on xylene coated glass-slides (Superfrost Plus™, Thermo Fisher Scientific, Germany), stained with 0.1% Toluidine blue after xylene deparaffinization, air-dried and mounted with DPX mounting medium (Honeywell Fluka, USA).

### Vegetative bud hormone quantification

The concentration of IAA, ABA, active GA (GA_1_ and GA_4_), and CKs (DHZ, iP, trans-zeatine tZ), and JA was determined in vegetative buds. 100 mg of frozen and ground vegetative buds were sent for analysis to Service of Hormone Quantification at IBMCP (Instituto de Biología Molecular y Celular de Plantas), Universidad Politécnica de Valencia, following the procedure reported in Methods S1.

### RNA sequencing (RNA-seq) and identification of differentially expressed genes (DEGs)

Total RNA was extracted from vegetative buds collected in Year A and from the vegetative and floral buds collected in Year B. RNA extraction was carried out using RNeasy Plant Mini kits from Qiagen following the manufacturer's instruction, with minor modifications as reported in Canton *et al*. [12]. RNA sequencing was conducted for year A vegetative buds collected at 0, 475, and 770CU and year B vegetative and flower buds collected at Pre0, 200, 475, and 930CU (Table 1).

Library preparation, sequencing, and data pre-processing were performed as reported in Methods S2. Data were normalized using TMM. Further statistical tests were conducted on NOISeq package with the normalized data (Fig. S2). The resulting list was further filtered using the Degust online platform (https://degust.erc.monash.edu/) with default parameters to filter out all genes with an overall fold change (FC) of less than 1.4.

Quantitative Real-Time PCR expression analysis was performed on specific target genes such as, PpeDAM6, PpeDAM5, PpeDAM4, PpeDAM3, PpeSVP, and PpeWDR5 following the protocol described in Canton *et al*. [12].

### Principal component analysis (PCA) of selected genes

Selected genes involved in hormone metabolisms (ABA, GA, JA) and meristem development were analyzed via a principal component analysis (PCA). Genes were firstly classified into flower-biased, vegetative-biased, or balanced categories based on a linear modeling approach, where Condition distinguishes flower vs vegetative buds, and Timepoint accounts for temporal variation. Linear regression models were fitted using the Linear Regression implementation from the scikit-learn library [53]. For details see Methods S3.

### Gene Co-expression network construction, network visualization, and module analysis

Weighted Gene Correlation Analysis was performed to group the DEGs into clusters based on their expression profile and draw correlations between the DEGs and the prevailing environmental conditions. For WGCNA and network inference, a more stringent filter (FC ≥ 1.5, FDR < 0.01) was applied to enhance module stability, differently from that used for the global DEG dataset (FC > 1.4; FDR < 0.05).The gene correlation networks were constructed with default parameters while providing the environmental conditions in the form of CU accumulation and LOD. Gene co-expression modules were identified using the WGCNA R package [54]. Hierarchical clustering, module assignment, and visualization of eigengenes and module-trait correlations were performed using standard WGCNA functions, including plotDendroAndColors, plotEigengeneNetworks, and labeledHeatmap. Functional profiling of gene clusters was conducted separately for flowerl and vegetative datasets. GO term enrichment was performed on modules V1–V3 (vegetative buds) and F1–F3 (flower buds) using the compareCluster function from the clusterProfiler R package (v4.12.6). Genes were annotated with *Prunus persica* v2 GO terms, and enrichment distributions were visualized comparatively across modules. Gene co-expression networks (GCNs) were independently constructed for vegetative and flower buds using filtered expression matrices restricted to genes assigned to relevant WGCNA modules (V1–V3 and F1–F2, respectively). For each dataset, pairwise Pearson correlations were computed between genes based on normalized expression values. To retain biologically meaningful interactions and reduce network complexity, only the five most highly correlated partners were retained per gene, and correlation values below *r* = 0.75 were discarded. The resulting adjacency matrices were used to generate undirected graphs using the NetworkX Python package. Nodes represent genes and edges correspond to high-confidence co-expression links. Genes not connected to any other were excluded from the network. Node degree was calculated and used as a proxy for centrality. Network visualization was performed using a Kamada-Kawai layout to enhance clarity.

### Gene regulatory network inference and visualization

Gene regulatory networks (GRNs) were inferred using the GENIE3 algorithm implemented in R. The expression matrix was constructed by merging floral and vegetative bud datasets and aligning genes based on their unique *Prunus persica* gene identifier (PRUPE_ID). Genes were then classified into three categories: shared genes, present and expressed in both organs; floral-specific genes, expressed exclusively in floral buds; and vegetative-specific genes, expressed exclusively in vegetative buds.

A curated list of DAMs- and SVP1, and 2 was used to define the set of candidate regulators for network inference. GENIE3 was executed using Random Forest (treeMethod = ‘RF’) with 500 trees per target gene (ntrees = 500). The resulting regulatory importance scores (‘weights’) were filtered to retain only high-confidence TF–target interactions (typically weight > 0.3). To focus on developmentally relevant interactions, target genes were further filtered for significant GO enrichment (adjusted *P* < 0.05). Final networks were reconstructed separately for shared, floral-specific, and vegetative-specific gene sets, allowing comparison of regulatory architectures across bud types. The GRN layout and graphical elements were generated using the NetworkX library (version 2.8.4) for network construction and Matplotlib (version 3.5.3) for rendering. Nodes were styled according to module assignment and GO annotation, while edges were scaled and colored based on GENIE3 regulatory weight, dimer type, and BiFC validation.

### Protein–protein interaction studies and BiFC assay

Candidate genes for interaction with PpeSVP were selected from vegetative bud DEGs based on similar or opposite expression patterns. Arabidopsis orthologs were screened using the ConnecTF platform to identify known interaction partners.

For BiFC analysis, full-length CDSs were amplified with attB-tagged primers and cloned into pDONR221 (ThermoFisher) vectors via BP recombination: SVP, DAM3, and DAM6 into P2P3; other genes into P1P4. Final constructs were assembled into the pBIFCt2in destination vector [55] by LR Clonase reactions (ThermoFisher). Positive (DAM6–DAM5) and negative (DAM3–DAM5) control pairs were included. Gene pairs and vector combinations are listed in Table S11.

BiFC constructs were introduced into Agrobacterium tumefaciens GV3101 and infiltrated into *N. benthamiana* leaves [56]. Fluorescence was assessed by confocal microscopy (YFP: 513 nm; RFP: 588 nm), with imaging support provided by the NO LIMITS advanced imaging facility at the University of Milan.

Predicted 3D structures from AlphaFold2 (Galaxy EU) were used in silico docking via ZDOCK (zdock.umassmed.edu), and interaction models were visualized with Jalview.

## Supplementary Material

Web_Material_uhaf310

## Data Availability

The data that support the findings of this study will be openly available in Gene Expression Omnibus at number GSE298924 for reference RNA-Seq data. Secure token for reviewer access: wjadiggqvfcrpez.
