# Supplementary material for: Beyond dormancy: organ-specific gene regulatory networks control winter development in peach buds
Source: Hortic Res. 2025 Nov 6;13(2):uhaf310. doi: 10.1093/hr/uhaf310 (PMC12936440; doi:10.1093/hr/uhaf310)
Supplement: Web_Material_uhaf310 [file web_material_uhaf310.zip › Supplementary Methods HR.docx]

**Methods S1** Vegetative bud hormone quantification

The finely ground tissue was suspended in 80% methanol-1% acetic acid containing internal standards and mixed by shaking for one hour at 4°C.The extract was stored at -20°C overnight and then centrifuged, with the supernatant being dried in a vacuum evaporator. The dry residue was dissolved in 1% acetic acid and passed through a reverse phase column (HLB Oasis 30 mg, Waters) as described in (Seo et al. 2011). For CKs, the extracts were additionally passed through an Oasis MCX (cationic exchange) and eluted with 60% methanol-5% NH4OH to obtain the basic fraction containing cytokinins. To recover the acid fraction, the MCX cartridge was eluted with methanol. The final residues were dried and dissolved in 5% acetonitrile-1% acetic acid, and the hormones were separated by UHPLC with a reverse Accucore C18 column (2.6 μm, 100 mm length; Thermo Fisher Scientific) using an acetonitrile gradient containing 0.05% acetic acid at 400 μL/min. For GAs and ABA, the gradient was 2 to 55% acetonitrile over 21 min.The Q-Exactive mass spectrometer (Orbitrap detector; ThermoFisher Scientific) was utilized to analyze the hormones in the extracts. Targeted Selected Ion Monitoring (tSIM) was used with a capillary temperature of 300ºC, S-lens RF level of 70, and a resolution of 70,000. Electrospray ionization was utilized in negative mode for acidic hormones or positive mode for CKs, with a spray voltage of 3.0 kV, heater temperature of 150ºC, sheath gas flow rate of 40 μL/min, and auxiliary gas flow rate of 10 μL/min. The concentrations of the hormones in the extracts were determined using embedded calibration curves and the Xcalibur 4.0 and TraceFinder 4.1 SP1 programs. The internal standards for quantification of each of the different plant hormones were deuterium-labelled hormones, which were purchased from OlChemim Ltd, Olomouc, Czech Republic. Statistical analysis was performed with R software. ANOVA was performed in order to identify the differences between the timepoints (cold effect), then when significant, Tukey test was applied with Bonferroni correction. Differences were considered significant at the p ≤ 0.05 level.

**Methods S2** Library preparation, sequencing and data pre-processing

For the vegetative buds, library preparation and sequencing using the Illumina system was performed by BMR Genomics Srl., Padua. Total RNA-seq was conducted for the vegetative buds collected during Year A for the three timepoints 0, 475 and 770 CU. Three replicates were sequenced for each time-point for both RNA-seq runs in vegetative buds. To maintain uniformity with the previous RNA-seq dataset from flower buds, only two replicates were sequenced for the flower buds in Year B. RNA-seq pre-processing was done on Galaxy Europe cloud bioinformatics portal (usegalaxy.eu). The sequenced reads underwent quality control and pre-processing using fastp v0.20.0 to remove adapter sequences and retain high-quality reads. The minimum read length was 15, with a phred score of 20; unqualified percent limit and complexity threshold were 30%. The filtered reads were mapped using STAR v2.7.0 with default parameters using the peach genome reference Prunus persica NCBIv2.51 (https://www.ncbi.nlm.nih.gov/genome/388?genome_assembly_id=312190). Annotations were made using references from the NCBI repository. To generate separate raw counts matrices from the alignment files for vegetative and flower buds were further elaborated using the Multicovbed bamcov function in bedtools version 2.30.1 with the following parameters: duplicated reads were discarded; only exons mappings were counted.to generate separate raw counts matrices from the alignment files for vegetative and flower buds. Further analysis using the raw counts were done using the R 4.2.1programming language. The multiple datasets for each of the two types of buds were initially merged. The merged datasets were pre-treated with the NOISeq package (Tarazona et al. 2011, 2015) to account for batch effects arising from the different sequencing runs and read biases, using the arsynseq function within the package with default parameters.

References

Tarazona S, García-Alcalde F, Dopazo J, Ferrer A. et al. Differential expression in RNA-seq: a matter of depth. *Genome Res*. 2021*,* **21**: 2213-23. https://org.doi/10.1101/gr.124321.111

Tarazona T, Furió-Tarí P, Turrà D. *et al.* Data quality aware analysis of differential expression in RNA-seq with NOISeq R/Bioc package. *Nucleic Acids Res.* 2015, **43**, ePage e140. https://doi.org/10.1093/nar/gkv711

**Methods S3**  Principal Component Analysis (PCA) of Selected Genes

Genes with p < 0.05 and a positive Condition coefficient were classified as vegetative-biased, while those with a negative coefficient were classified as floral-biased. Genes with non-significant differences (p ≥ 0.05) were classified as balanced. To reduce false classification due to variability, genes with |Δ_VF| ≤10% of the maximum expression value between organs were also considered balanced. PCA was conducted only on shared genes, i.e. those expressed in both floral and vegetative buds. Expression values across six time points per organ were standardized using z-score transformation (mean-centered and scaled to unit variance). Organ-specific genes (detected in only one organ type) were excluded from PCA calculation but projected post hoc into the same PC space using the loadings obtained from the shared genes. PCA and projection analyses were performed in Python using scikit-learn (version 1.3.2), and the figures were created using Matplotlib (version 3.8.2). Pathway categories (biosynthesis, catabolism, conjugation, perception, signaling) were used to color-code gene symbols. Specific genes are shown in bold font, and shared genes in regular font.

Gene ID from Prunus persica v2 (PRUPE) were converted with the Arabidopsis thaliana ortholog gene name. To perform this task, a double strategy was applied:

- Gene ID from Prunus were directly converted to orthologs Arabidopsis gene name through the Plant Biomart (https://plants.ensembl.org/biomart/martview)

- Gene names not detected with the previous approach were identified through Phytozome v13 extracting the matching GeneID descriptions.

- For the DAM gene ids were converted to those reported in literature (Canton et al., 2021).

References

Canton M, Forestan C, Bonghi C. et al. Meta-analysis of RNA-Seq studies reveals genes with dominant functions during flower bud endo- to eco-dormancy transition in Prunus species. *Sci. Rep*. 2021; **11**. doi:10.1038/s41598-021-92600-6.
