# Supplementary material for: Beyond dormancy: organ-specific gene regulatory networks control winter development in peach buds
Source: Hortic Res. 2025 Nov 6;13(2):uhaf310. doi: 10.1093/hr/uhaf310 (PMC12936440; doi:10.1093/hr/uhaf310)

Supporting Information

Article title: **Beyond dormancy: organ-specific gene regulatory networks control winter development in peach buds**

Authors: Justin Joseph, Giorgio Perrella, Riccardo Aiese Cigliano, Marco di Marsico, Monica Canton, Esther Carrera, Lucio Conti, Claudio Bonghi and Serena Varotto

The following Supporting Information is available for this article:

Fig. S1 Quantification of trans-zeatine (tZ), isopentenyladenine (iP), Cytokinins dihydrozeatin (DHZ), Indol-3-Acetic acid (IAA), and GA1 in vegetative buds during chilling accumulation.

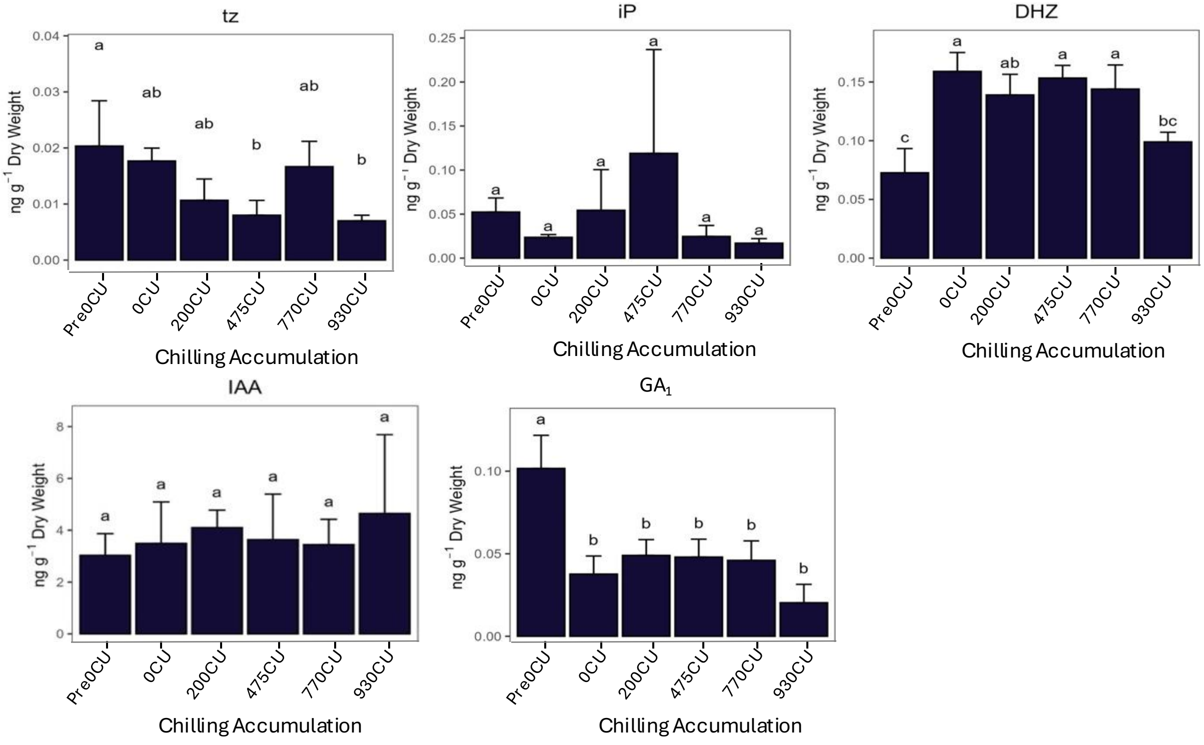

Fig. S2 Normalization of the multiple datasets from the different RNAseq experiments and removal of batch effect. Box plots a, e and b, f represent the distribution of counts among the different samples before and after normalization respectively in vegetative and floral buds. On the other hand, graphs c, g and d, h represent principal component analysis of the different samples from the two bud types respectively before and after removal of the batch effect.

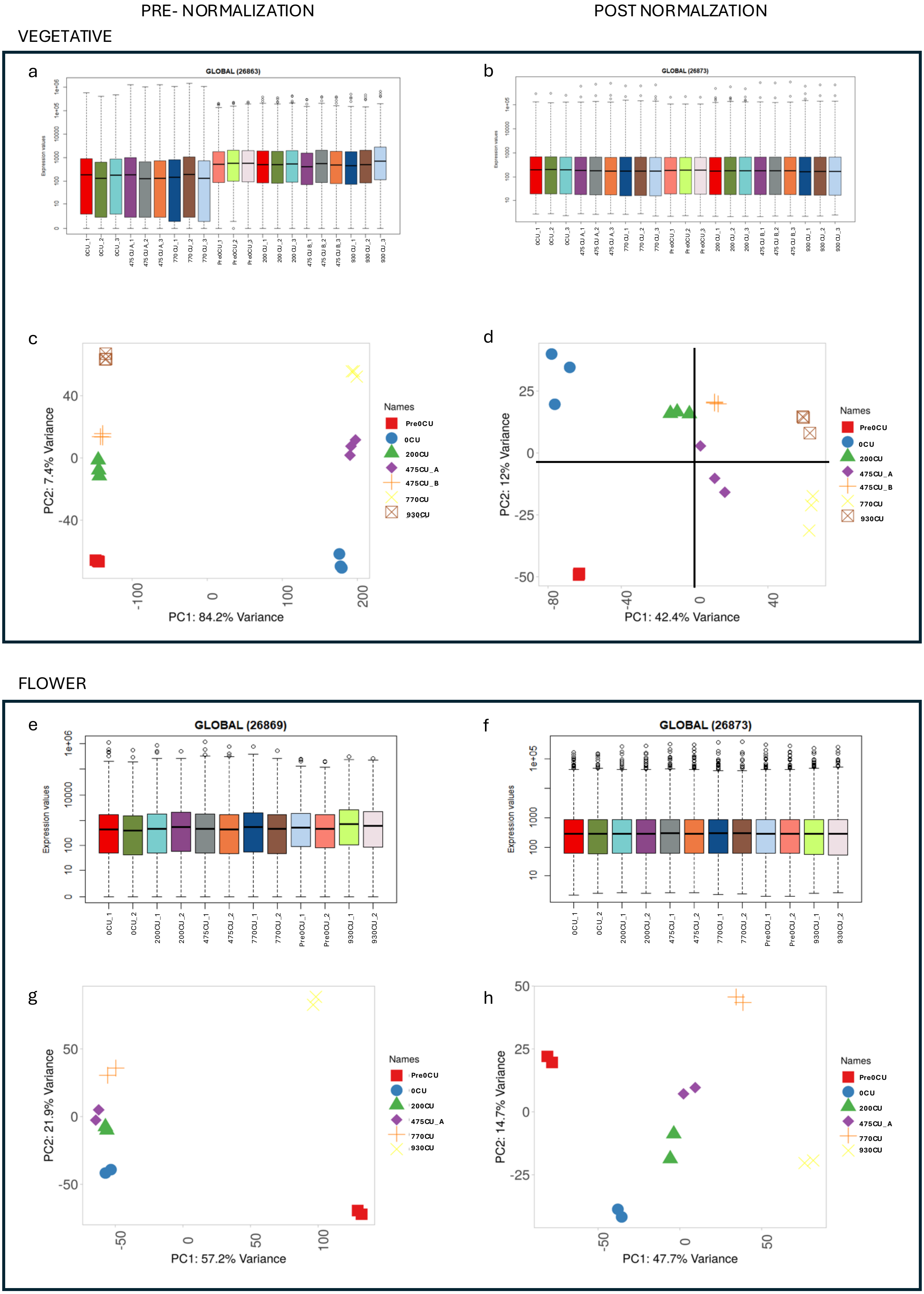

Fig. S3. Differential Express Genes in vegetative and flower buds. A Progressive pairwise comparison between the different timepoints against PreCU0 in vegetative and flower buds, respectively. B, Venn diagrams showing common DEGs across the different timepoints in vegetative and flower buds.

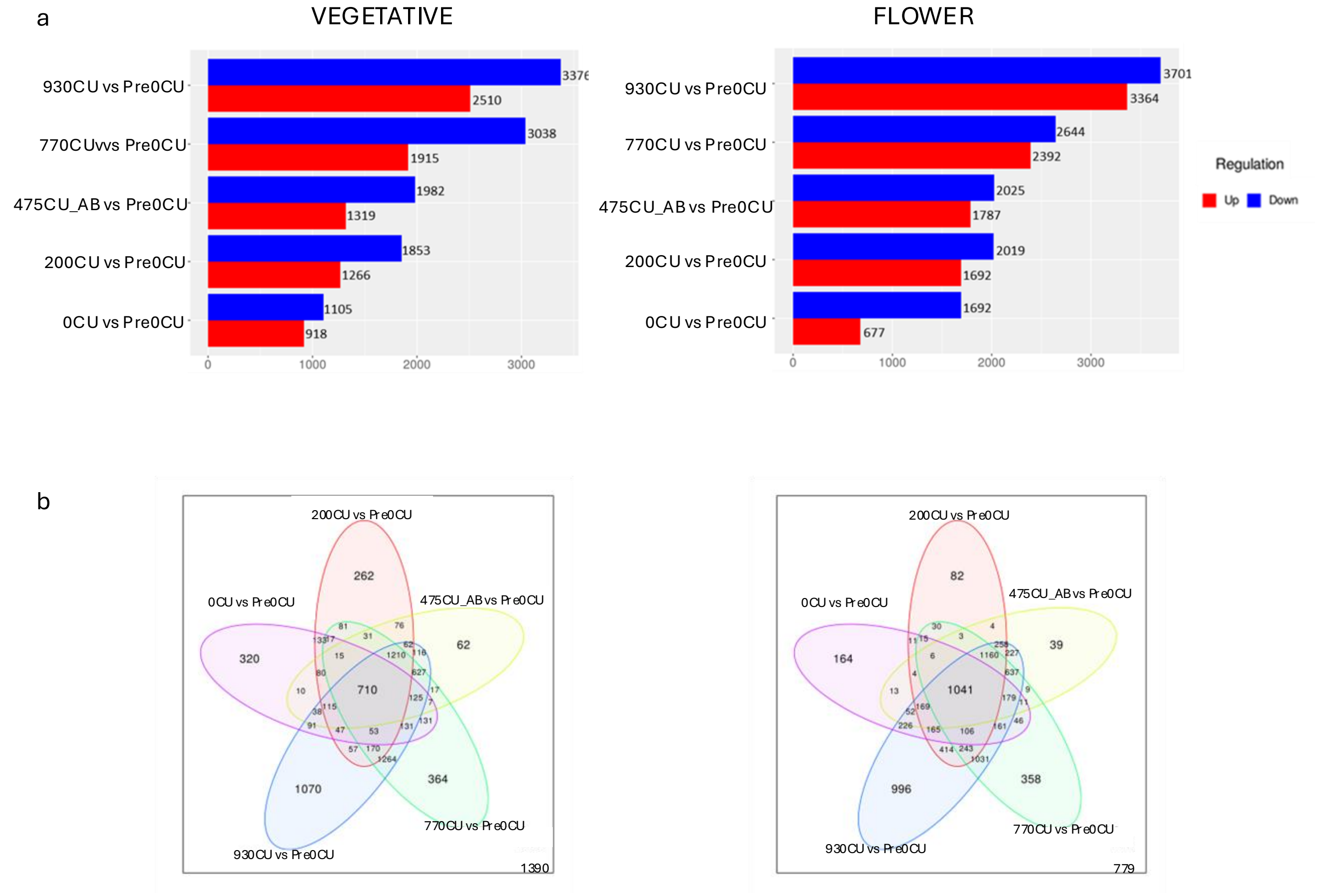

Fig. S4. Measurements of length of day across the sampling period. Visibly there is little variation between the length of day over the three sampling periods between 2019 and 2023.

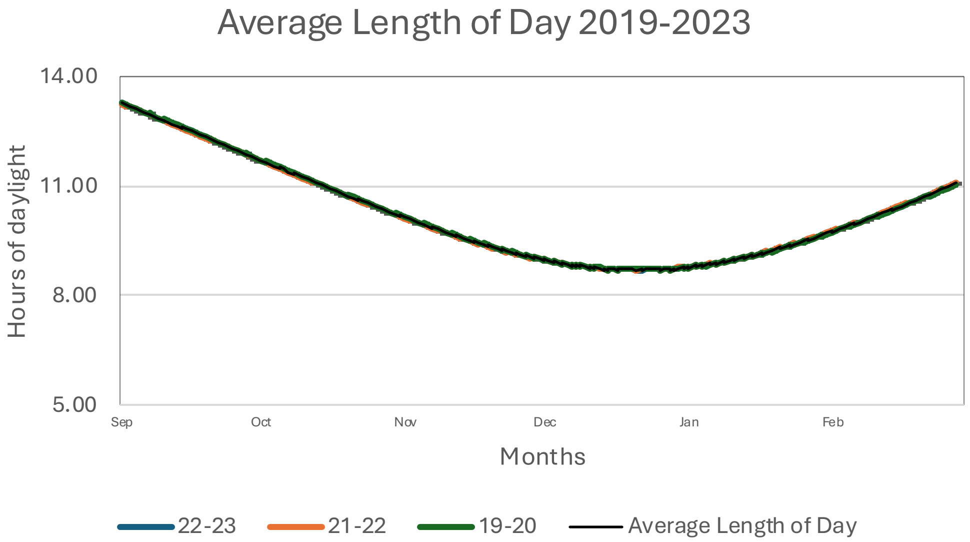

Fig. S5. BiFC assay results. Each panel displays the YFP and RFP channel of a gene pair. The RFP channel forms the internal control for transformation. Nuclear fluorescence highlighted with an arrow in the YFP channel denotes a positive interaction. a & b represent the positive (DAM5-DAM6) and negative (DAM6-DAM3) controls. Panels c, d and e represent the positive interactions between SVP-DAM3, SVP-DAM5 and SVP-DAM6 respectively. The remaining gene pairs which did not interact with SVP are f - PILS7, g – CIB8, h- PIF8, i – AP22, j - ERF1, k – ERF2, l- AUX/IAArep

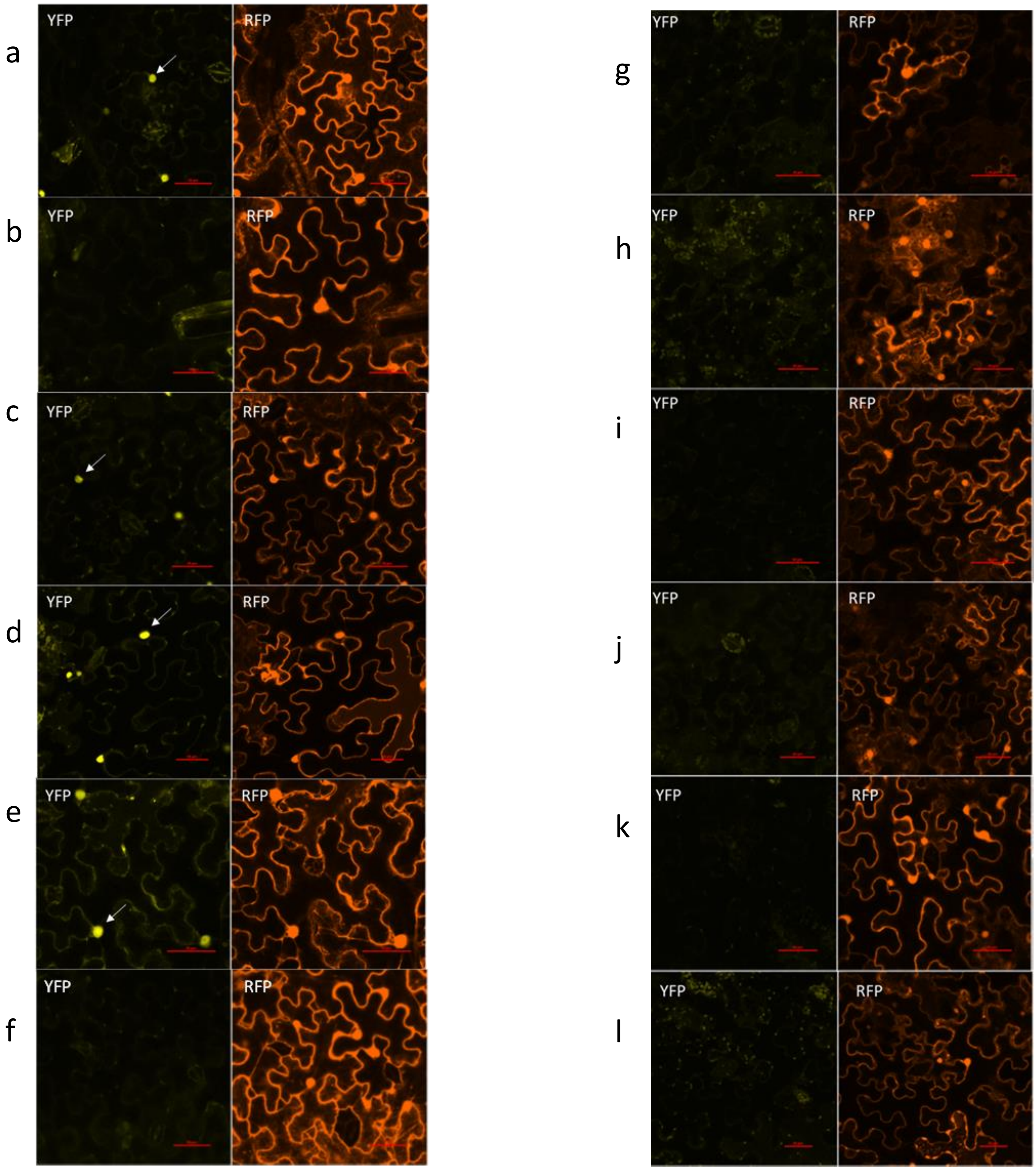

Fig. S6. Distribution of regulatory weights inferred by GENIE3 for floral, vegetative, and shared gene targets. Histogram showing the distribution of regulatory importance scores (weight) assigned by GENIE3 to target genes regulated by DAM/SVP transcription factors in flower (yellow), vegetative (orange), and shared (red) regulatory networks.

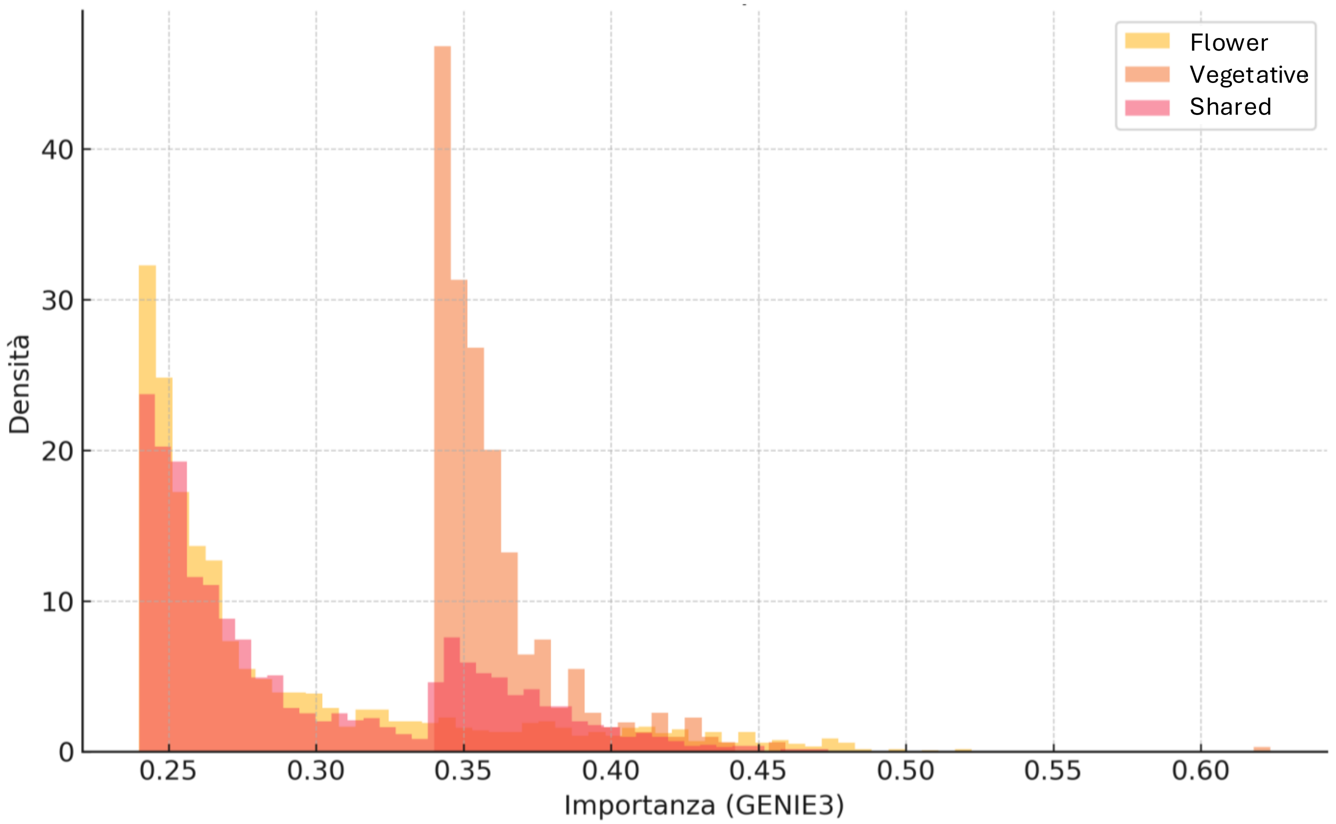

Fig. S7. Gene regulatory networks (GRNs) predicted targets for DAM and SVP-like transcription factors in flower peach buds. Nodes represent target genes (circles, squares, triangles) or transcriptional regulators (orange pentagons). Node shapes and colors indicate WGCNA module membership: light blue circles (F1) and light green squares (F2). Node borders denote Gene Ontology (GO) categories: grey (developmental process), purple (multicellular organism development), orange (glucose metabolism) and red (flower development initiation). Asterisks (*) indicate genes annotated in multiple GO categories. Only targets with significant GO annotation are shown; unannotated targets were omitted for clarity. Edges represent regulatory interactions inferred by GENIE3: solid black (homodimer-based) and orange (heterodimer-based regulation). Edge thickness reflects GENIE3 importance scores: thin (<0.3, weak), medium (0.3–0.5, moderate), thick (>0.5, strong). Full interaction data and GO assignments are provided in Table S9.

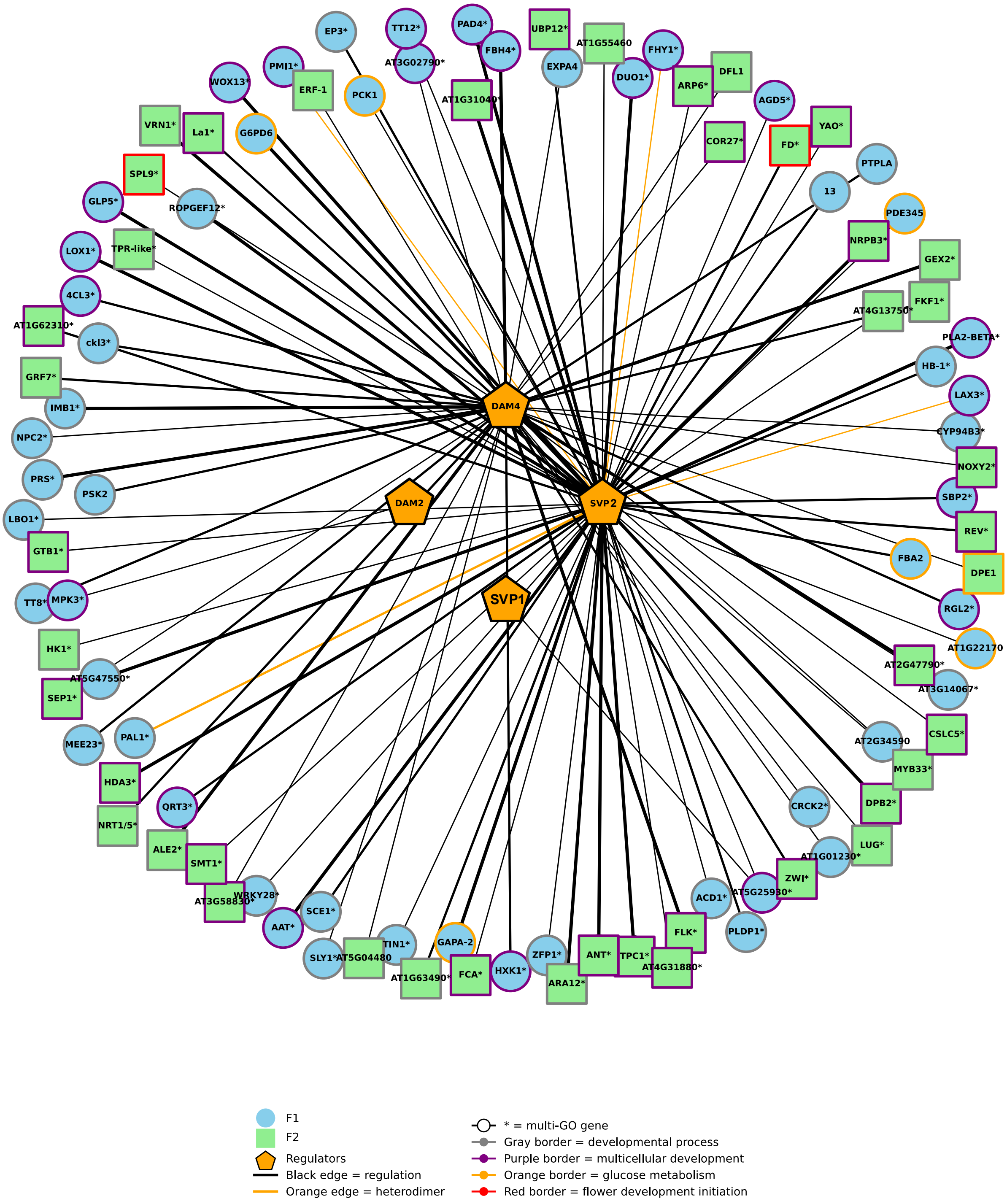

Fig. S8. Gene regulatory networks (GRNs) predicted targets for DAM and SVP-like transcription factors in vegetative peach buds. Nodes represent target genes (circles, squares, triangles) or transcriptional regulators (orange pentagons). Node shapes and colors indicate WGCNA module membership: light blue circles (V1), light blue squares (V2), and light red triangles (V3). Node borders denote Gene Ontology (GO) categories: black (developmental process), purple (post-embryonic development), and light green (plant organ development). Asterisks (*) indicate genes annotated in multiple GO categories. Only targets with significant GO annotation are shown; unannotated targets were omitted for clarity. Edges represent regulatory interactions inferred by GENIE3: solid black (homodimer-based), orange (heterodimer-based regulation), and blue (BiFC-validated physical interactions). Edge thickness reflects GENIE3 importance scores: thin (<0.3, weak), medium (0.3–0.5, moderate), thick (>0.5, strong). Full interaction data and GO assignments are provided in Table S10.

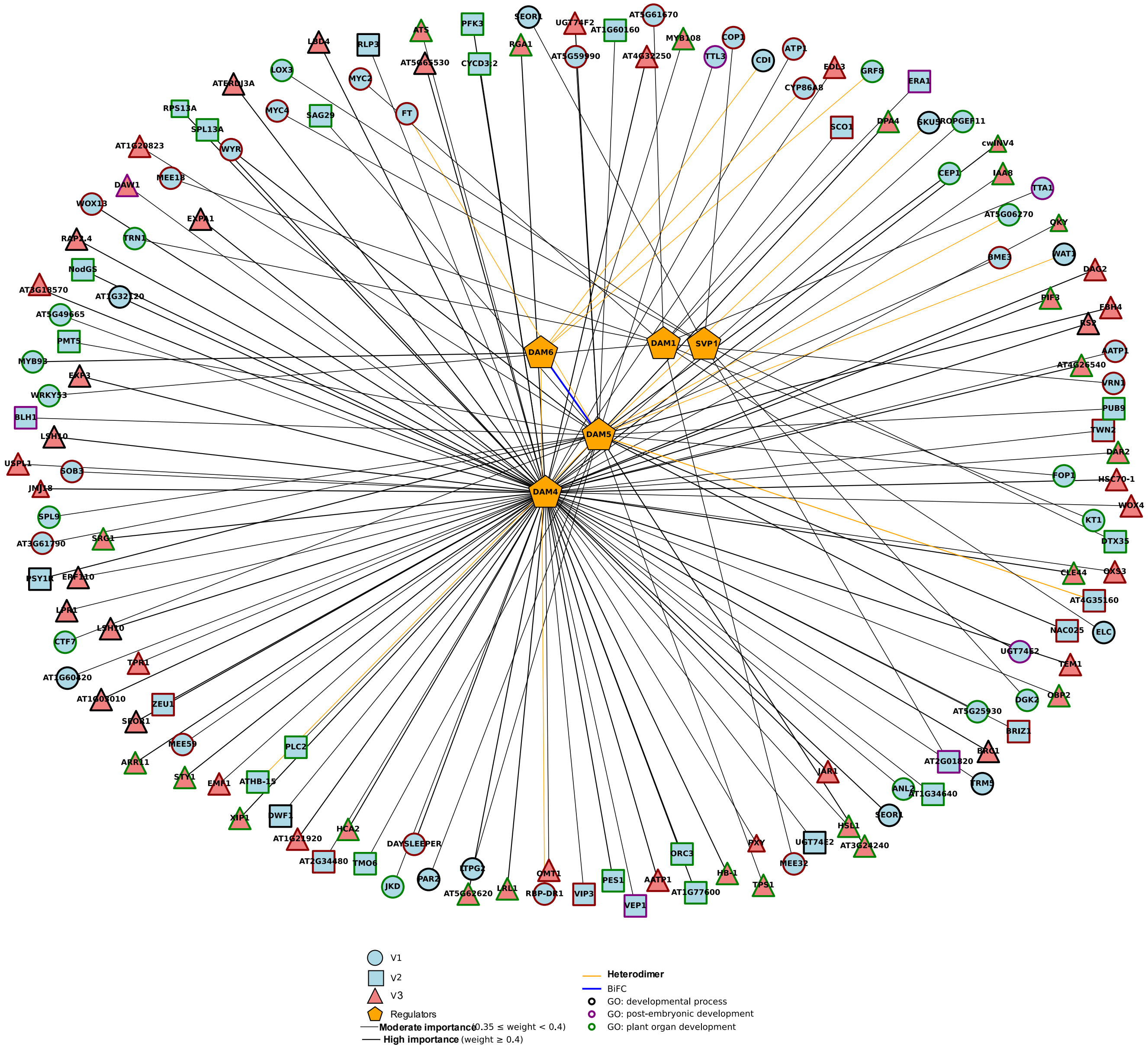

Supplement: Web_Material_uhaf310 [file web_material_uhaf310.zip › Supporting Figures HR.pdf]
